# Prediction of Force Measurements of a Microbend Sensor Based on an Artificial Neural Network

**DOI:** 10.3390/s90907167

**Published:** 2009-09-09

**Authors:** Hasan S. Efendioglu, Tulay Yildirim, Kemal Fidanboylu

**Affiliations:** 1 Fatih University, Department of Electrical and Electronics Engineering, 34500 Buyukcekmece, Istanbul, Turkey; E-Mail: kfidan@fatih.edu.tr (K.F.); 2 Yildiz Technical University, Department of Electronics and Communications Engineering, 34349 Yildiz, Istanbul, Turkey; E-Mail: tulay@yildiz.edu.tr (T.Y.)

**Keywords:** artificial neural networks, fiber optic sensors, microbend sensors, multilayer perceptron, radial basis function, general regression neural network

## Abstract

Artificial neural network (ANN) based prediction of the response of a microbend fiber optic sensor is presented. To the best of our knowledge no similar work has been previously reported in the literature. Parallel corrugated plates with three deformation cycles, 6 mm thickness of the spacer material and 16 mm mechanical periodicity between deformations were used in the microbend sensor. Multilayer Perceptron (MLP) with different training algorithms, Radial Basis Function (RBF) network and General Regression Neural Network (GRNN) are used as ANN models in this work. All of these models can predict the sensor responses with considerable errors. RBF has the best performance with the smallest mean square error (MSE) values of training and test results. Among the MLP algorithms and GRNN the Levenberg-Marquardt algorithm has good results. These models successfully predict the sensor responses, hence ANNs can be used as useful tool in the design of more robust fiber optic sensors.

## 1. Introduction

Fiber optic sensors offer several advantages compared to conventional sensors. First, fiber optic sensors can easily be integrated into structures due to their small size and cylindrical geometry, forming what is called smart structures. They are also all-dielectric and immune to electromagnetic interference. Moreover, they are lightweight, robust and have large bandwidths. In addition to these advantages, improvements and cost reductions in fiber optic technology have also stimulated interest in fiber optic sensors in the last decade.

Microbend sensors were among the earliest fiber optic sensors developed and have been employed by some researchers for sensing of many parameters since the 1980s. Typical applications include sensing of acoustic and displacement [1], pressure, acceleration, magnetic and electric fields [2], temperature [3] and pH [4].

When a fiber is subjected to small deformations (microbends), light rays in the core of the fiber exceed the critical angle. This causes redistribution of energy between core and cladding modes. The guided higher order core modes couple to the cladding modes causing the light propagating in the fiber to decrease. This mode coupling can be achieved by placing the fiber in contact with a series of periodically positioned deformers. Hence, microbending causes the light intensity to decrease due to light leakage into the cladding. By monitoring and correlating the loss of light intensity, different types of microbend sensors can be designed [5]. In addition to the general advantages of fiber optic sensors, microbend sensors are easier to implement and have lower cost than other types of fiber optic sensor [6].

Artificial Neural Networks (ANNs) have become a popular artificial intelligence technique due to their ability to learn and fast real-time operation. They have been extensively investigated as a computational paradigm in which a deterministic description of the computation is either difficult to identify or too complex [7,8]. These characteristics made ANNs useful in optical fiber technology for the prediction of measurements of fiber optic sensors [9], development of intelligent fiber optic sensors [10], signal processing [11,12] and calibration [13].

Researchers have also used ANNs in fiber optic sensor design in the following areas: recovery of information about strain and temperature from fiber optic sensors [14], signal processing of optical fiber pH sensor based on bromophenol blue doped with sol-gel film [15], pattern recognition in a three sensor multipoint optical fiber water sensor [16], pattern recognition in an optical fiber ethanol concentration sensor [17] and humidity estimation [18].

In this paper, we have used the normalized intensity as a function of applied force obtained from a microbend sensor to predict the response of the sensor by using ANNs since these can generate appropriate outputs for given inputs without any necessity for mathematical formulations between input and output data. To the best of our knowledge, no similar work has been presented in the literature.

## 2. Overview of Artificial Neural Networks

Artificial Neural Networks (ANNs) are neuron-like processing elements which try to mimic simple nervous systems. An artificial neuron model comprises input(s) with weight(s), activation function(s) and output(s). The weights are adjusted until the desired output is generated for a given input. Several types of activation functions can be used in applications. Hyperbolic tangent and sigmoidal functions are among the activation functions commonly used. ANNs are mainly used for classification, function approximation, clustering and regression.

ANNs have different types of connections. Feed forward neural networks are the most popular model, used in many applications. They are also called Multi Layer Perceptron (MLP) networks. A MLP generally consists of an input layer, one or more hidden layers and an output layer. The processing units are arranged in layers [19].

Radial Basis Function (RBF) is another network model which is a class of hybrid connection model. Whilst they are essentially three-layer feed forward networks, RBF networks differ from classical MLP in three significant ways: there is only one set of trainable weights, from the hidden layer to the output layer; the nodes’ activation functions are non-standard and learning is affected by both supervised and unsupervised techniques [20].

The General Regression Neural Network (GRNN), which is a kind of radial basis network, is a powerful regression tool with a dynamic network structure. The network training speed is extremely fast. It is simple and can be easily implemented [20].

There are many training algorithms in the literature, so it is very difficult to know which training algorithm will be the fastest or more accurate for a given problem, as this depends on several factors, including complexity of the problem, the number of weights and biases in the network, the number of data points in the training set, the error goal and the task the network is to be used for [19]. The algorithms used to train MLP in this work are Gradient Descent with Adaptive Learning Rate Backpropagation (DA), Resillient Backpropagation (RB), Fletcher-Reeves Update (FRU), Polak-Ribiere Conjuage Gradient (CGP), Conjugate Gradient with Powell/Beale Restarts (CGB), Scaled Conjugate Gradient (SCG), Broyden-Fletcher-Goldfarb-Shanno (BFGS), and Levenberg-Marquardt (LM).

The DA is a technique in which the learning rate is allowed to change during the training process. With standard steepest descent, the learning rate is held constant throughout training. The purpose of the RB method is to eliminate the harmful effects of the magnitudes of the partial derivatives. Only the sign of derivative is used to determine the direction of the weight update; the magnitude of the derivative has no effect on the weight update. The size of the weight is determined by a separate update value. The FRU is a network training function that updates weights and bias values according to the conjugate gradient backpropagation with Fletcher-Reeves updates. It has the smallest storage requirements of the conjugate gradient algorithms. The CGP is a network training function that updates weight and bias values according to the conjugate gradient backpropagation with Polak-Ribiere updates. The CGB is a network training function that updates weight and bias values according to the conjugate gradient backpropagation with Powell/Beale Restarts. The SCG is a member of the class of the conjugate gradient method is the only conjugate gradient algorithm that requires no line search. The BFGS is a quasi-Newton method which is an alternative to the conjugate gradient methods for fast optimization. The LM is a network training function that updates weight and bias values according to the Levenberg-Marquardt optimization. The LM algorithm was designed to approach second order training speed without using a Hessian matrix [8,19].

## 3. Microbend Sensor Measurements and Experimental Procedure

**Figure 1.**
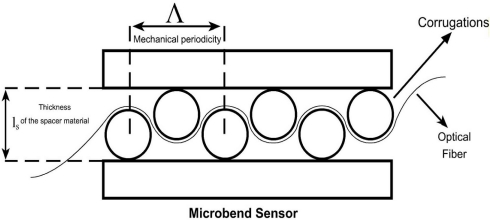
The geometry of the sensing region.

The geometry of a sensing region of a microbend sensor is shown in Figure 1. The sensing region consists of two corrugated plates. The corrugations are cylindrical rods with fixed diameters. The fiber is pressed between these plates by applying different forces to the top plate. Optical fiber passes through these corrugated plates. Both ends of the fiber inside the sensor are relaxed to keep away elastic factors of the fiber. Λ is the mechanical periodicity (deformer tooth spacing), *l_s_* is the corrugation size (thickness of the spacer material between the deformer plates) and cylindrical rods are the corrugations (bends).

Intensity modulation caused by microbending in multimode fibers can be exploited as a transduction mechanism for detecting environmental changes. The change in light transmission, Δ*T*, through a microbend sensor can be formulated as:

(1) $$\Delta T=\left(\frac{\Delta T}{\Delta X}\right)\;\Delta F\;{\left(k_{f}+\frac{A_{s}\;Y_{s}}{l_{s}}\right)}^{-1}$$

where ΔX is the deformer plates’ displacement, Δ*T*/ Δ*X* is the sensitivity, ΔF is the applied force, *A_s_* is the area, *l_s_* is thickness of spacer, Y_s_ is the Young’s modulus, and the *k_f_* is the effective spring constant of the optical fiber [2]. The effective spring constant can be expressed as follows:

(2) $$k_{f}^{-1}=\frac{\Lambda^{3}}{3\pi {\mathrm{Yd}}^{4}\eta }$$

where *Y* is the effective Young’s modulus, *d* is the diameter of fiber and *η* is the number of bent intervals [9].

From Equations (1) and (2), it can be seen that if a force applied to the microbend sensor (ΔF), the intensity of the light at the output of the fiber will change. In addition, the geometrical parameters of the deformer such as mechanical periodicity, deformer cross sectional area, deformations spacing and number of corrugations will affect the output intensity of light. Since microbend sensors fall into the group of intrinsic fiber optic sensors, the fiber properties such as Young’s modulus and diameter of the fiber will affect the output intensity too.

**Figure 2.**
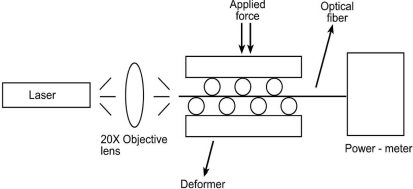
Schematic of experimental setup.

**Figure 3.**
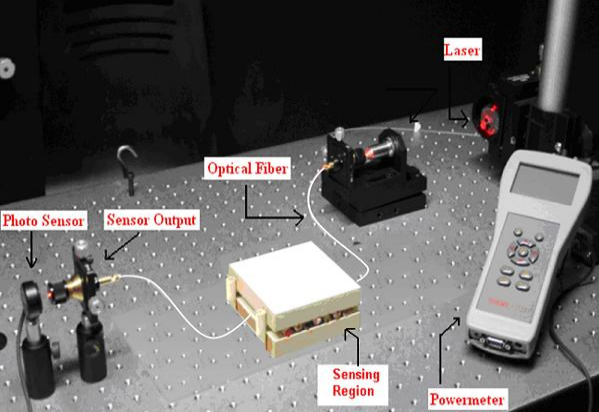
Experimental setup.

To understand the behaviour of fiber optic microbend sensors, an experiment was conducted. The optical intensity was measured as a function of applied force to the microbend deformer set. The experimental setup consisted of a 16 mW solid-state laser with a 650 nm wavelength, a step index multimode silica fiber with a numerical aperture (NA) of 0.37, and core/cladding/jacket dimensions of 200/230/500 μm, microbend deformer and a power-meter. Schematic and picture of the experimental setup are shown in Figures 2 and 3, respectively. Light obtained from a solid state laser and coupled into the multimode fiber with a 20X objective lens. All the sensors had three deformation cycles. Experiment was conducted by increasing force from 0 N to 58.66 N. The working range of this experiment is between 0.98 N and 83.385 N. For each applied force, the output light intensity was measured by using photodetector. The deformer spacing was 12 mm and the mechanical periodicity was 16 mm with three deformation cycles.

**Figure 4.**
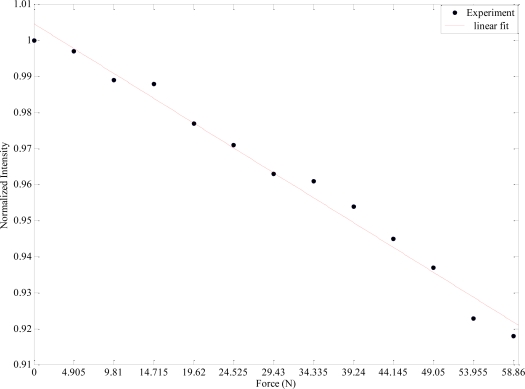
The sensor response (normalized intensity) in terms of F.

Figure 4 shows the results obtained from this experiment. From this figure it can be observed that as the applied force increases, the output light intensity decreases. The amount of light loss while increasing the force is an important parameter for sensitivity in fiber optic microbend sensors since the magnitude of the measurand can be determined by correlating the loss of light intensity.

## 4. Application of ANN to Microbend Sensor and Results

MLP with different algorithms (RBF and GRNN) have been used to estimate the microbend sensor response. The input and output variables of ANN models are force (F) and normalized intensity, respectively. The general structure of the networks proposed in this paper is shown in Figure 5.

F is a known parameter and can be utilized for the prediction of the sensor response. A training dataset consisted of randomly selected F vs. normalized intensity is exploited to the network and the network is trained. After the training process, new responses are predicted for unseen F values.

Thirteen samples were used in the network. Eight of these samples were used in the training dataset. The performance of the algorithms used in the network is compared in terms of their mean square errors (MSEs). The training algorithms and the number of hidden neurons used in MLP network are given in Table 1. The training MSEs results of MLP are given in Table 2. These results show that the LM algorithm has the smallest MSE value, even though it has one of the smallest numbers of neurons.

The training MSEs results of RBF and GRNN are given in Table 3. From the results it can be inferred that RBF and GRNN algorithms have small MSE values in comparison with the different MLP algorithms. However, among the algorithms RBF has the best performance with the smallest MSE value.

The prediction performances of different ANN models proposed in this work have been tested with five experimental data sets obtained from the fiber optic microbend sensor described in the experimental procedure in Section 2. The comparisons of the sensor responses and the ANN model outputs are given in Table 4. From this comparison it can be perceived that RBF has the smallest MSE value. The worst MSE value belongs to BFGS. The best (RBF) and the worst (BFGS) ANN outputs with respect to MSEs are graphically compared with the experimental results in Figure 5.

## 5. Conclusions

Different ANN approaches have been proposed in this work to predict the response of a fiber optic microbend sensor. First it can be said that ANN approaches can tolerate measurement errors of fiber optic microbend sensors. Performance comparisons demonstrate that all of the ANN models used in this paper can predict the sensor responses with considerable errors. RBF has the best performance with the smallest MSE values of training and test results. GRNN and Levenberg-Marquardt algorithm among the MLP algorithms also have good results. These models successfully predict the sensor responses. From this work it can be concluded that the proposed technique is a useful tool in the design of more robust fiber optic sensors.

## Figures and Tables

**Figure 5.**
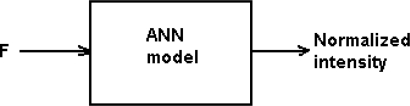
General structure of the networks proposed in this work.

**Figure 5.**
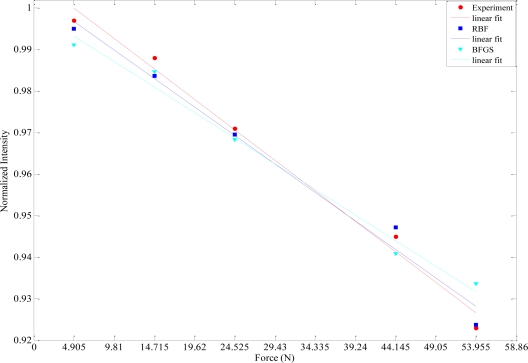
Comparisons of the best and the worst ANN outputs with the sensor responses.

**Table 1.** The training algorithms and number of hidden neurons in MLP.

**Algorithm**		**Number of Hidden Neurons in MLP**

MLP	DA	6
	RB	6
	FRU	5
	PRU	4
	CGB	4
	SCG	3
	BFGS	5
	LM	4

**Table 2.** The MSEs of different training algorithms for MLP.

**Algorithm**		**Mean Square Error (MSE)**

MLP	DA	1.9320E-07
	RB	1.7005E-07
	FRU	1.7415E-09
	PRU	5.9327E-09
	CGB	1.9657E-07
	SCG	1.0030E-06
	BFGS	5.2094E-09
	LM	8.2732E-16

**Table 3.** The MSEs of RBF and GRNN

RBF	4.1600E-32
GRNN	3.1917E-20

**Table 4.** Comparison of the sensor responses and the ANN outputs.

**F(N)**	**Sensor Response**	**ANN Model Outputs**									
		**DA**	**RB**	**FRU**	**PRU**	**PBR**	**SCG**	**BFGS**	**LM**	**RBF**	**GRNN**
4.905	0.997	0.9956	0.9944	0.9912	0.9915	0.9912	0.9950	0.9912	0.9917	0.9951	0.9945
14.715	0.988	0.9800	0.9839	0.9846	0.9861	0.9859	0.9829	0.9847	0.9862	0.9837	0.9830
24.525	0.971	0.9687	0.9649	0.9693	0.9667	0.9673	0.9701	0.9683	0.9664	0.9696	0.9700
44.145	0.945	0.9507	0.9452	0.9409	0.9429	0.9444	0.9488	0.9409	0.9424	0.9472	0.9455
53.955	0.923	0.9263	0.9261	0.9336	0.9319	0.9294	0.9244	0.9336	0.9321	0.9237	0.9275
	**MSE**	**2.2926E-05**	**1.4086E-05**	**3.5452E-05**	**2.7194E-05**	**1.8612E-05**	**9.4440E-06**	**3.6198E-05**	**2.8412E-05**	**5.8780E-06**	**1.0550E-05**
